# Perpetrator as a Potential Victim. Does Threatened Retaliation from the Victim Reduce Obedience towards Authority?

**DOI:** 10.5334/pb.397

**Published:** 2017-07-03

**Authors:** Tomasz Grzyb, Dariusz Doliński

**Affiliations:** 1 SWPS University of Social Sciences and Humanities, Faculty of Psychology in Wroclaw, PL

**Keywords:** retaliation, social influence, obedience, Milgram paradigm

## Abstract

In an experiment conducted within the Milgram paradigm, it was examined whether obedience towards an authority would be reduced in conditions in which the teacher had grounds to fear revenge from the learner. A comparison was made of the behaviour of participants in classic conditions and in conditions in which they were told that following the first part of the experiment, there would be an alteration of roles: the teacher would become the learner. It turned out that the level of compliance was the same in both groups. The dominant behaviour, regardless of whether the participant expects a change of roles or not, is total obedience.

Stanley Milgram’s demonstration (1963, 1965) that the majority of people will agree to administer a 450-volt electric shock to another person, if such an instruction is given to them by a professor from a respected university conducting an experiment allegedly exploring the effectiveness of punishment on learning efficiency, shocked both the scientific community and public opinion.

Stanley Milgram explained the obedience of his experiments’ participants by invoking an agentic state understood as one in which “the individual no longer views himself as responsible for his own actions but defines himself as an instrument for carrying out the wishes of others” (Milgram, 1974, p. 134). The subject literature, however, contains other suggestions as to what may have led to such results being generated. An interesting reinterpretation of the behaviours of experimental participants in the Milgram paradigm was offered by Gilbert (1981). He suggested evaluating the Milgram experiment from the perspective of the classic social influence technique known as foot-in-the-door (Freedman & Fraser, 1966). The essence of this technique consists in boosting the chances that the addressee will fulfil a difficult request by first issuing an easier one. An individual who fulfils the first, easier request will then be more inclined to meet a successive, more difficult one, particularly if it is similar in substance to the initial request. In Milgram’s experiments, the participant is first asked to shock the “learner” with 15 volts of electricity. If the participant agrees to give a shock of 15 volts, he will then agree to press the second button (only 15 volts stronger than the preceding one). He then consents to press successive buttons, because the difference between what he is supposed to do and what he has just done is minimal. We are thus dealing with a phenomenon which can be termed ‘multiple foot-in-the-door’, a highly effective technique as has been thoroughly demonstrated in experiments on social influence (e.g. Goldman, Creason, & McCall, 1981; Seligman, Miller, Goldberg, Geldberg, Clark, & Bush, 1976). While such an interpretation of the behaviours of individuals participating in experiments using the Milgram paradigm has not been demonstrated empirically yet (Dolinski and Grzyb, 2016), it is worth observing that it does help to understand the phenomenon of the birth and growth of totalitarian movements. For example, the fact that during World War II the Germans committed heinous crimes on such a large scale is easier to understand when considering that at the beginning they were encouraged to paint the Star of David on the window of a Jewish storefront. Once they had done this, it was easier to convince them to throw a stone through that window, and later to progress to acts of increasing indignity and brutality, culminating in consent to the murder of humans and direct, active involvement in the process.

Remaining with the subject of the behaviour of Nazis supporters during the Hitler era, it is worth drawing attention to one particular fact concerning the birth of anti-Nazi movements. They were practically non-existent in Germany at the time when Hitler was enjoying his greatest successes, then grew in strength following the collapse of the German army at Stalingrad. This was the time when White Rose, Kreisau Circle, and other informal organizations came about. Although historians generally explain this by stating that Hitler’s military success made it difficult to find approval for the idea of removing him from power or murdering him (e.g. Fest, 1996; Scholl, 1983), it could alternatively be claimed that the disaster at Stalingrad and subsequent failures induced a segment of Germans to think that Germany could evolve from a country occupying the other nations of Europe to one occupied by its former victims. This could have given rise to the obvious question of “How will people who were previously victims behave towards their former oppressors?” The decision of some to act and put a stop to Nazi terror meted out towards people of other nationalities could have been, at least in part, motivated by fear over the fate of their homeland, as well as of themselves and their loved ones.

Numerous psychological studies demonstrate that not only victims are frequently inclined to seek revenge (e.g. Aquino, Tripp & Bies, 2006; Konig, Gollwitzer, & Steffgen, 2010; Tripp, Bies & Aquino, 2007; Skarnicki & Folger, 1997), but also that their actions are frequently viewed to be justified, as they aim at achieving justice. In the eyes of society, victims thus have the right to seek revenge in accordance with the ancient rule of “an eye for an eye, a tooth for a tooth” (e.g., Govier, 2002; Gollwitzer, 2009). It is worth noting that in the classic Milgram experiments (1974), the participant could always operate under the assumption that the learner being shocked with electricity would not have an opportunity to take revenge. What would happen, however, if the “teacher” was convinced that he would later become the learner, and the person who had previously been the learner would then be in the position to make decisions about shocking him? Would this lead to thoughts such as “He will have the right of revenge. If I zap him now, he will zap me in the future. If I refuse to zap him, he will also refuse to zap me”?

This thinking could also be substantiated from the perspective of the well-known reciprocity principle (Cialdini, 2006; Falk & Fischbacher, 2006; Goren, Bornstein, 1999; Uehara, 1995). While the vast majority of experiments concerning the reciprocity principle are focused on the norm of returning favours, pleasantries or presents (and thus on things that are generally positive), there are also examples of analyses going in the opposite direction (Gouldner, 1960; Chen et al., 2009). The so-called negative norm of reciprocity assumes that from the social perspective, those who inflict harm on others should be harmed proportionally to what they themselves have done (Eisenberger, Lynch, Aselage, & Rohdieck, 2004; Perugini, Gallucci, Presaghi, & Ercolani, 2003). We may thus expect that individuals “zapping with electricity” the “learner” sitting in the other room will be inclined to think that they will get the learner’s revenge when their roles are to be reversed, and will therefore be more inclined to refuse to press subsequent buttons, even in the face of pressure from an authority figure.

While this issue has been explored in psychology (Constanzo, 1976), it was done so as part of an experiment that was to serve as the basis of a PhD dissertation, and its results have never been published in any journal. The experiments were conducted at the University of Wyoming, and 96 students of that institution participated. Constanzo explored such issues as how the threat of revenge impacted upon obedience among study participants. After presenting participants with instructions for how to proceed in the course of the experiment, it was added (in the group with the retaliation condition) that after a series of guessing stimuli and electric shocks, the participants will change roles and the teacher will become the learner. The results attained demonstrated that the threat of revenge did not have a significant effect on the obedience exhibited by study participants. In conditions of potential revenge, 7 of 48 participants refused to continue the experiment, while in the control group 11 of 48 participants did so. However, it should be added that in Constanzo’s experiments, the procedure itself was different from that of Milgram. The participants were told that “the task will involve mental telepathy in which one of you, the sender, concentrates for five seconds on the suit of a card and tries to transmit the image of that suit to the receiver. The receiver, in turn, will try to pick up that image”. In addition, while in the Milgram studies it was necessary to resist on four occasions to finish participation, with Constanzo it was sufficient for the participant to refuse three times. The fact that the experiment’s participants were only students (and thus representatives of a rather unique population), who did not receive any financial gratification (distinguishing it from the classic Milgram experiments) should also be noted. All of these factors may have exerted a real influence on the results recorded. It is also worth emphasizing that four decades have passed since Constanzo’s experiments, and both the passage of time and historical events could have markedly modified people’s readiness to follow the instructions of an authority figure as well as the likelihood of them of being afraid of revenge.

Studies conducted in the Milgram paradigm as referenced multiple times in this article are, on the one hand, incredibly important for psychology; on the other hand, they have been a source of ethical dilemmas from the very beginning (e.g. Fisher, 1968; Kaufmann, 1967; Mixon, 1972). While in the 1970s there were some replication experiments conducted in various countries around the world (e.g. Bock and Warren, 1972; Kilham and Mann, 1974; Shanab and Yahya, 1978), further experiments in that paradigm have not been performed. A few years ago Burger (2009) conducted an experiment in which he applied the Milgram paradigm in a manner that minimized the discomfort felt by study participants. Most importantly, due to ethical concerns he asked participants to press only 10 (and not 30, as in the original) buttons. The procedure proposed by Burger was also used in later studies (Dolinski & Grzyb, 2016; Dolinski et al, 2017).

In our experiment we decided to compare the functioning of people tested in “classic” conditions of the Milgram procedure (when the participant had no grounds to suspect revenge from the “learner”) with that of people who find out that during the first phase they will be teachers, and thus shocking the learner with electricity after every mistake, while in the second phase they will switch roles. The teacher would thus become the learner and would be zapped with electricity every time a mistake was made. It can be assumed that in conditions in which participants could expect that revenge would be taken, they would be less compliant; this would result in a refusal to comply with the experimenter’s instructions. At the same time, for ethical reasons, we decided to conclude the study at the moment when the participant pressed the 10th button on the generator, marked with a label reading 150 volts (as Burger, 2009, did).

## Method

### Participants

Recruitment was conducted in a very similar manner to that of Milgram’s studies. A local newspaper published on multiple occasions an advertisement of around 1/3–1/2 page, proposing participation in psychological experiments linked with learning and memorizing. For 45 minutes of their time in a laboratory, they were promised a sum of PLN 50 (approx. USD 12.5). Those interested in the offer were asked to make contact by e-mail or telephone. Screening was then conducted. Students were disqualified, as were individuals who had taken a psychology course when they were students. Potential participants were also asked if they had heard of any psychological experiments, and those whose answers indicated that they may have been exposed to any information at all regarding Milgram’s experiments were also eliminated. Three more people were removed in the course of the study proper. One of them (a woman) demonstrated a very strong fearful reaction and disorientation when the workings of the equipment were demonstrated to her; the second (a man) arrived inebriated; the third (a man), after being shown the device for administering electric shocks, said that he had seen a film on the internet about such studies. The three eliminated individuals were replaced by others. A total of 40 people participated in the experiment, with 10 women and 10 men in each condition; participants were within the age range of 21–57 years (*M* = 38.77; *SD* = 10.27).

### Procedure

Participants were randomly assigned to one of two experimental conditions (classic conditions in which the participant was given the role of teacher) and “role reversal”, in which the participant was told that he would be the teacher during the first phase, and the learner in the second. Care was taken to ensure the same number of men and women participated in each condition.

Our experiment was based on experiment no. 2 described in “Obedience to authority: An experimental view” (Milgram, 1974). While Experiment 5, described in the same work, is widely regarded as the standard for demonstrating obedience, distinctly from Experiment 2 it is far more burdensome on those in the experiment (owing to the information that the learner has heart trouble). Because our objective was not the simple replication of Milgram’s study, but only to test a hypothesis on the role of the revenge motive, such an approach was optimal in our view.

Participants came to the office of the professor conducting the experiment at one of the university buildings in one of Poland’s largest cities, where they were greeted by him. The experimenter was a white male, over 50 years of age, dressed formally in a suit and tie, with a badge containing his picture, name, and title of professor fixed to his jacket. After a moment the confederate arrived, who explained that he had come for the psychological experiment. The confederate was a white male aged around 35 years, dressed casually in jeans and a t-shirt.

The experimenter thanked the participants for coming and asked them to complete a consent form. In these forms it was written that participants would be paid PLN 50 in advance, and that they could resign from further participation in the study at any moment without forfeiting the financial gratification. After both individuals had signed the declaration, the experimenter paid them (in cash) PLN 50 each, and again emphasized that regardless of the further course of the experiment and any potential decision to resign, the money was already theirs. He then explained that the experiment concerned the impact of punishments on memorization and learning, and that the procedure required assuming roles. In the classic condition, he said that one person would serve as the teacher in the experiment, while the other would be the learner. The experimenter placed two pieces of paper on a table in front of the participants; each of them had the word “teacher” written on it. This meant that the “real” participant declared he had just selected that role, while the confederate claimed he had drawn the card with the word “learner”. In the role reversal conditions, the experimenter explained that during the first phase of the experiment one person would be the learner and would then become the teacher, while the other would first be the teacher and then the learner. “In other words”, he summarized, “after the first phase, you will switch roles” and placed two pieces of paper before the participants. Each of them contained the words “first teacher, then learner”, which meant that the “real” participant had “randomly selected” that role, while the confederate announced he had randomly selected the role “first learner, then teacher”.

The trio then proceeded to the laboratory, where the experimenter showed the electricity generator to the participant and confederate (designed exactly the same as the apparatus used by Milgram), and explained that the learner’s role was to consist in learning by rote associations between certain syllables. For example, after hearing “BA” he was supposed to respond “BO”, and after hearing “DA” the response should be “DE”. The experimenter handed a piece of paper with eight pairs of syllables to the confederate, explained that he now had some time to learn those pairs, then led him to a neighbouring room. He then returned and gave the teacher a list of 45 pairs of syllables written down in a different order than on the paper given to the learner; next he explained to the teacher that his task would consist in reading one of the syllables and waiting for the learner’s response. If the response was correct, he should proceed to reading the next syllable. An incorrect response meant waiting for instructions from the experimenter. They would address the issue of the punishment to be administered to the learner. The experimenter explained that these instructions would involve administering an electric shock. He then demonstrated the workings of the machine, showing the “teacher” that pressing particular buttons would activate a jolt of between 15 and 450 volts. He also read aloud information concerning the level of pain and damage to health that could arise from various levels of electrical intensity. To demonstrate the operation of the machine, he mounted electrodes on the subject’s wrist and pressed the third button, administering a shock of 45 volts (pilot studies demonstrated that the vast majority of people zapped with this level of electricity described their experience as aversive, but not painful). Next, the experimenter showed the teacher that there was another cable running from the generator through the wall to the neighbouring room where the “learner” was already sitting. He went to that room together with the “teacher” and, in his presence, mounted the electrodes to the wrist of the “learner”. Next, he took the learner’s paper with the sets of syllables and returned with the teacher to the room where the generator was located. An intercom system facilitated two-way verbal communication between the two laboratory rooms in which the study was carried out. The experimenter asked the participant to sit by the generator, while he himself sat around three metres from it, looking from the side. He then instructed the participant to read the first syllable. The learner responded properly, and the participant then read the next syllable. The learner did not make a mistake in this or in successive cases, but did err after the seventh syllable.

The experimenter then instructed the teacher to press the first button. The “learner” reacted from behind the wall with a grunt. The experimenter issued an instruction to read the next four syllables. The “learner” responded properly. However, he made a mistake at the tenth syllable. The experimenter told the teacher to press button number 2, and the learner reacted with a louder grunt. The next mistake came with syllable 13. The learner’s reaction in this case to the teacher pressing the (now third) button was a grunt. Successive impulses of energy (after successive mistakes at attempts number 15, 18, 22, 24, 27, 31 and 34) were associated with the learner crying out in pain. These screams were of increasing clarity and intensity. If the teacher wavered, or expressed doubt as to the use of electricity on the learner, the experimenter implored him to do so using an analogical exhortation to those in Milgram’s experiments (1974): “Please continue”, “The experiment requires that you continue”, “It is absolutely essential that you continue”, “You have no other choice, you must go on”.

Upon completion of the study a very thorough debriefing was held. First, it was established whether the participants were indeed convinced they were administering an electric shock to the person sitting behind the wall (apart from one previously mentioned man with whom the experiment was interrupted at an earlier stage, none of the participants had any doubt that they were in fact doing so), and then the details of the experimental procedure were explained; this was followed by an apology for the deception. Participants were informed that in such experiments, the majority of people go so far as to press the final button; at the same time they were told that the experience may serve them as a life lesson, that – at least sometimes – it may be too easy to succumb to pressure exerted by others. Any and all questions from the participants were also answered, and they were encouraged in the event of further doubts or questions to contact the experimenters by telephone or e-mail.

### Results

The experiment was conducted in a 2 × 2 format (sex of participants vs. experimental conditions). In the classic conditions, 3 of 20 participants (2 women and one man) refused to press all 10 buttons (one quit the study when the experimenter tried to convince her to press button number 4, the second refused to press button number 7, while the third refused to press button 8). The remaining 17 people pressed all buttons, and in only two cases was it necessary to implore a wavering participant with the message “Please continue”. In conditions of expected role reversal, 2 of 20 participants refused to press button number 10 (one of them was a woman, the other a man, both of whom refused to press button number 8). In respect of the remaining 18 people, only twice did the experimenter invoke the exhortation “Please continue”. Sex had no impact on the results.

The difference in the proportion of people in the two experimental conditions who turned out to be entirely obedient was statistically insignificant (two-tailed Fisher exact probability test: *p* = 1; *phi* = .08), but it should be emphasized that obedience was by far the dominant behaviour in both conditions we tested (total obedience occurred in the case of 35 of 40 participants, 87.5%).

### Discussion

In the experiment we examined whether the obedience noted on multiple occasions in studies conducted under the Milgram paradigm would be limited in conditions where the participant expected to soon become the recipient of an electric shock following each mistake. Contrary to our expectations, it turned out that also in these conditions the clearly dominant behaviour of participants was total obedience towards the experimenter, following all of his instructions. Although ethical considerations led to us limiting the number of participants to 20 in each of the conditions, it is worth emphasizing the very small difference in the behaviour of participants in the two conditions. Both in “classic” and “role reversal” conditions nearly all of the participants pressed the successive buttons on the generator, and only in a few cases did the experimenter need to resort to additional verbal exhortations contained in the experiment plan. This result is particularly interesting in light of studies on aggression, which have demonstrated that fear of revenge on the part of the victim leads to a clear drop in the tendency of the potential aggressor to do harm (Baron, 1971, 1973, 1974; Rogers, 1980). McCullough, Kurzban, and Tabak (2013) even perceive this mechanism as one of the fundamental evolutionary conditions that led our ancestors on the one hand to be less aggressive towards other people, while on the other hand making it easier to establish close relationships. Milgram conducted a study in which participants could independently decide about punishing the learner, and only 2.5% of participants inflicted every shock. If there was some innate aggressive drive in humans, when participants had the option to inflict shocks of great intensity surely more than 2.5 percent of them would have inflicted every shock. Nevertheless, some researchers continue to suggest that if people can justify their innate aggression (e.g. through pressure from an authority figure, as in the classic Milgram studies), they will display it (see for example the discussion of this issue in Blass, 2004). Because the results pattern we have recorded is entirely different from that which was generated in studies on aggression, we have by the same token acquired yet another powerful argument in favour of the theory that the behaviour of participants in the Milgram experiments is an example of their obedience and servitude, rather than aggression.

However, it should be kept in mind that we cannot entirely exclude desire on the part of study participants to withdraw from the experiment just prior to shifting from the role of the teacher to that of the learner and vice-versa. As we have previously explained, participants were clearly informed of that right (both in writing and verbally) prior to the commencement of the experiment. It is thus easy to imagine that a portion of them intended to perform all of the experimenter’s instructions (shocking the learner with electricity), and then invoke their right to withdraw from the experiment following the reversal of roles. While this behaviour would run contrary to the widely-known rule of engagement and consistency (e.g. Becker, 1960; Cialdini, 2006), it cannot be entirely excluded. It should be kept in mind, however, that empirical verification of this fact would have to consist in really shocking the participants with electricity, which is impossible due to ethical considerations. A partial solution would be to conduct the experiment up to the moment at which the former teacher is connected to the device as the learner, and begin the procedure anew; however, it may be supposed that a significant number of participants would attempt to endure at least the initial weak electric shocks.

In offering the hypothesis that fear of revenge may reduce obedience, we have invoked the example of anti-Hitler movements, which began to appear when German society started coming to terms with the potential consequences of the downfall of the Third Reich. It should be noted, however, that such movements were exceedingly marginal and consisted only of a very small number of people. Obedience towards the leader remained the overwhelming universal and dominant mode. It would seem that we have recorded a similar result in our study. The strength of the fear of revenge that was to induce people to refuse carrying out the experimenter’s orders turned out to be too weak in the face of those forces that led people to display obedience.
